# Data of the COVID-19 mRNA-Vaccine V-Safe Surveillance System and Pregnancy Registry Reveals Poor Embryonic and Second Trimester Fetal Survival Rate. Comment on Stuckelberger et al. SARS-CoV-2 Vaccine Willingness among Pregnant and Breastfeeding Women during the First Pandemic Wave: A Cross-Sectional Study in Switzerland. *Viruses* 2021, *13*, 1199

**DOI:** 10.3390/v13081545

**Published:** 2021-08-05

**Authors:** Serge Stroobandt, Roland Stroobandt

**Affiliations:** 1 Independent Researcher, Kolonel Dusartplein 10, 3500 Hasselt, Belgium; serge@stroobandt.com; 2 Vaccination Center Oostende-Bredene, 8400 Oostende, Belgium

Dr. Sarah Stuckelberger and her colleagues should be commended for their cross-sectional study assessing the willingness of Swiss pregnant and breastfeeding women to be vaccinated against SARS-CoV-2 [1]. They emphasise the need to identify and reduce barriers towards immunisation. Furthermore, they express the hope that when more data become available about vaccinated pregnant women, willingness to be vaccinated will increase. Moreover, the authors refer to a study [2] that has been unequivocally heralded as a proof of safety for the use of COVID-19 mRNA vaccines in pregnant women.

Regardless, we would like to advise readers that the referenced article contains a serious error regarding the interpretation of the data presented in Table 4. Prospective cohort studies are routinely performed to establish the safety of novel obstetric interventions. Such studies typically compare, at the same gestational age, the wellbeing of a cohort that underwent the intervention to that of a comparable control cohort (or, in the absence of this, the pertaining population) without the intervention. Nonetheless, the cited study compared the control population’s incidence rate of spontaneous abortions of 10 to 26% prior to week 20 to the incidence among the 827 study participants of which 700 received their first dose only in the third trimester, i.e., after week 26. However, a correct comparison with the remaining 127 participants sets the 104 spontaneous abortions recorded prior to week 20 at an alarming incidence of 82%, i.e., 3 to 8 times higher than in the control population. This observation suggests that obstetric vaccine safety is severely compromised during pregnancy and may lead to decreased willingness among pregnant women to be vaccinated.

## Data Availability

Not applicable.
